# New variants of CRISPR RNA‐guided genome editing enzymes

**DOI:** 10.1111/pbi.12736

**Published:** 2017-05-09

**Authors:** Jana Murovec, Žan Pirc, Bing Yang

**Affiliations:** ^1^ Biotechnical Faculty University of Ljubljana Ljubljana Slovenia; ^2^ Department of Genetics Development and Cell Biology Iowa State University Ames IA USA

**Keywords:** genome editing, CRISPR effector C2c2, CRISPR/Cas9, Cpf1, variants of *Streptococcus pyogenes* Cas9, plant biotechnology

## Abstract

CRISPR‐mediated genome editing using the *Streptococcus pyogenes* Cas9 enzyme is revolutionizing life science by providing new, precise, facile and high‐throughput tools for genetic modification by the specific targeting of double‐strand breaks in the genome of hosts. Plant biotechnologists have extensively used the *S. pyogenes* Cas9‐based system since its inception in 2013. However, there are still some limitations to its even broader usage in plants. Major restrictions, especially in agricultural biotechnology, are the currently unclear regulatory status of plants modified with CRISPR/Cas9 and the lack of suitable delivery methods for some plant species. Solutions to these limitations could come in the form of new variants of genome editing enzymes that have recently been discovered and have already proved comparable to or even better in performance than *S. pyogenes* CRISPR/Cas9 in terms of precision and ease of delivery in mammal cells. Although some of them have already been tested in plants, most of them are less well known in the plant science community. In this review, we describe the following new enzyme systems engineered for genome editing, transcriptional regulation and cellular imaging—C2c2 from *L. shahii*; Cas9 from *F. novicida*,* S. aureus*,* S. thermophiles, N. meningitidis*; Cpf1 from *F. novicida*,* Acidaminococcus* and *Lachnospiraceae*; nickase, split, enhanced and other Cas9 variants from *S. pyogenes*; catalytically inactive *Sp*Cas9 linked to various nuclease or gene‐regulating domains—with an emphasis on their advantages in comparison with the broadly used *Sp*Cas9. In addition, we discuss new possibilities they offer in plant biotechnology.

## Introduction

**Figure 1:**
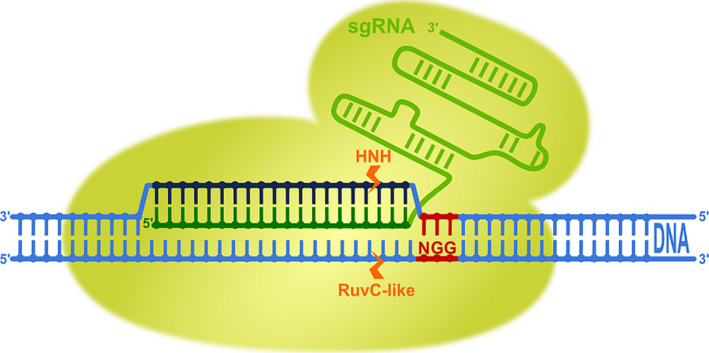
Genome editing through programmable RNA‐guided DNA endonuclease cleavage with type II CRISPR/Cas9 system from *Streptococcus pyogenes*, the Cas nuclease most commonly used. The two‐component system consists of Cas9, an helicase and endonuclease enzyme, and of a single guide RNA molecule, which was engineered from the dual tracrRNA:crRNA. sgRNA retains both critical features: the 20‐nucleotide‐long specific sequence at the 5′ end (dark green) and stem–loop RNA structure at the 3′ end (light green), which is needed for binding to the Cas9 enzyme. On assembly of sgRNA and Cas9, the complex first recognizes the dinucleotide protospacer adjacent motif sequence (5′‐NGG‐3′, PAM, red) of DNA and unwinds it to enable Watson–Crick RNA–DNA pairing of complementary bases between target DNA, the so‐called protospacer (dark blue) and the 20‐nt guide sequence of sgRNA (dark green). Double‐strand cleavage is performed by two Cas9 nuclease cleavage domains: the HNH domain cleaves the DNA strand that is complementary to the guide sequence of the sgRNA and the RuvC‐like domain cleaves the DNA strand opposite the complementary strand, both three bases upstream (orange arrows) of the PAM sequence.

Clustered regularly interspaced short palindromic repeats (CRISPR) is a ribonucleoprotein (RNA/protein)‐based adaptive immune system widespread in bacteria and archaea. It relies on two small RNAs (CRISPR RNA—‘crRNA’ and transactivating CRISPR RNA—‘tracrRNA’), which recognize invading foreign nucleic acids (in the forms of viruses, phages and plasmids) and in conjunction with CRISPR‐associated proteins (Cas) destroy them through site‐specific cleavage (Wiedenheft *et al*., 2012). CRISPR loci were first identified in *Escherichia coli* genome in 1987 as a series of 29‐bp‐long direct repeats interspaced with 32‐bp‐long spacer sequences (Ishino *et al*., 1987). Later, similar short partially palindromic repeat sequences of 24–40 bp were discovered in various physiological and phylogenetic groups of bacteria and archaea. These repeat sequences were conserved in members of the same phylogenetic group and highly similar even among domains. Between the repeats, unique intervening sequences of 20–58 bp were found (Mojica *et al*., 1995, 2000). In 2002, species‐specific repeat sequences of 21–37 bp were reported again, and a common sequence GTT and AAC was discovered at their ends. The locus was named clustered regularly interspaced short palindromic repeats (CRISPR) for the first time, and homologous CRISPR‐associated (*cas*) genes were discovered in the vicinity of this locus (Jansen *et al*., 2002). It was first hypothesized (Makarova *et al*., 2006) and later confirmed (Barrangou *et al*., 2007; Garneau *et al*., 2010) that CRISPR provides acquired resistance against pathogens in prokaryotes. A major breakthrough occurred in 2012, with the fusion of CRISPR RNA (crRNA) and transactivating CRISPR RNA (tracrRNA) in a single guide RNA (sgRNA) molecule (Figure 1), which accelerated the implementation of the CRISPR/Cas system in practice (Jinek *et al*., 2012). In 2013, the first publications described the use of the natural type II CRISPR/Cas system from *Streptococcus pyogenes* (*Sp*Cas9, Figure 1) for induction of site‐specific double‐strand breaks and subsequent mutagenesis in human, mouse and plant cells (Cho *et al*., 2013; Cong *et al*., 2013; Feng *et al*., 2013; Jiang *et al*., 2013; Jinek *et al*., 2013; Li *et al*., 2013; Mali *et al*., 2013; Miao *et al*., 2013; Nekrasov *et al*., 2013; Shan *et al*., 2013; Upadhyay *et al*., 2013; Xie and Yang, 2013). Since then, it has already been used in model, as well as commercially important, plant species such as *Arabidopsis*, tobacco, rice, wheat, maize, potato, tomato, soybean, sorghum, poplar, flax and many others. Induction of site‐specific DNA breaks in plants have been used for gene knockouts, deletions of chromosomal fragments, gene knockins, gene corrections, gene replacements and development of virus resistance in plants. Overviews about CRISPR/*Sp*Cas9 principles, targets, delivery methods and issues with off‐target mutations in plants have recently been published in various excellent reviews (Mei *et al*., 2016; Song *et al*., 2016; Sun *et al*., 2016; Weeks *et al*., 2016; Zhang *et al*., 2017) and are therefore not the topic of this review.

**Figure 2:**
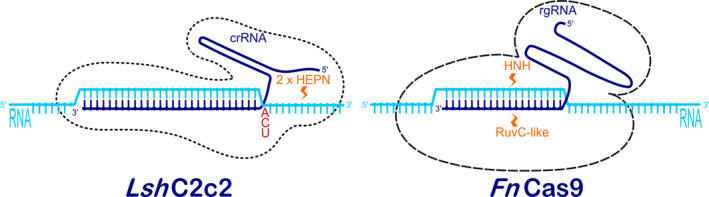
RNA‐guided RNase enzyme systems C2c2 from *Leptotrichia shahii* (left) and Cas9 from *Francisella novicida* (right). Target ssRNAs (light blue) are recognized by complementary regions of crRNA or rgRNA (dark blue) in *Lsh*C2c2 and *Fn*Cas9, respectively. In *Lsh*C2c2, cleavage is performed downstream of the protospacer‐flanking site (A, C or U, red) by two HEPN domains (orange), while in *Fn*Cas9 cleavage occurs within the complementary region via a combined activity of HNH and RuvC‐like nuclease domains (orange).

**Figure 3:**
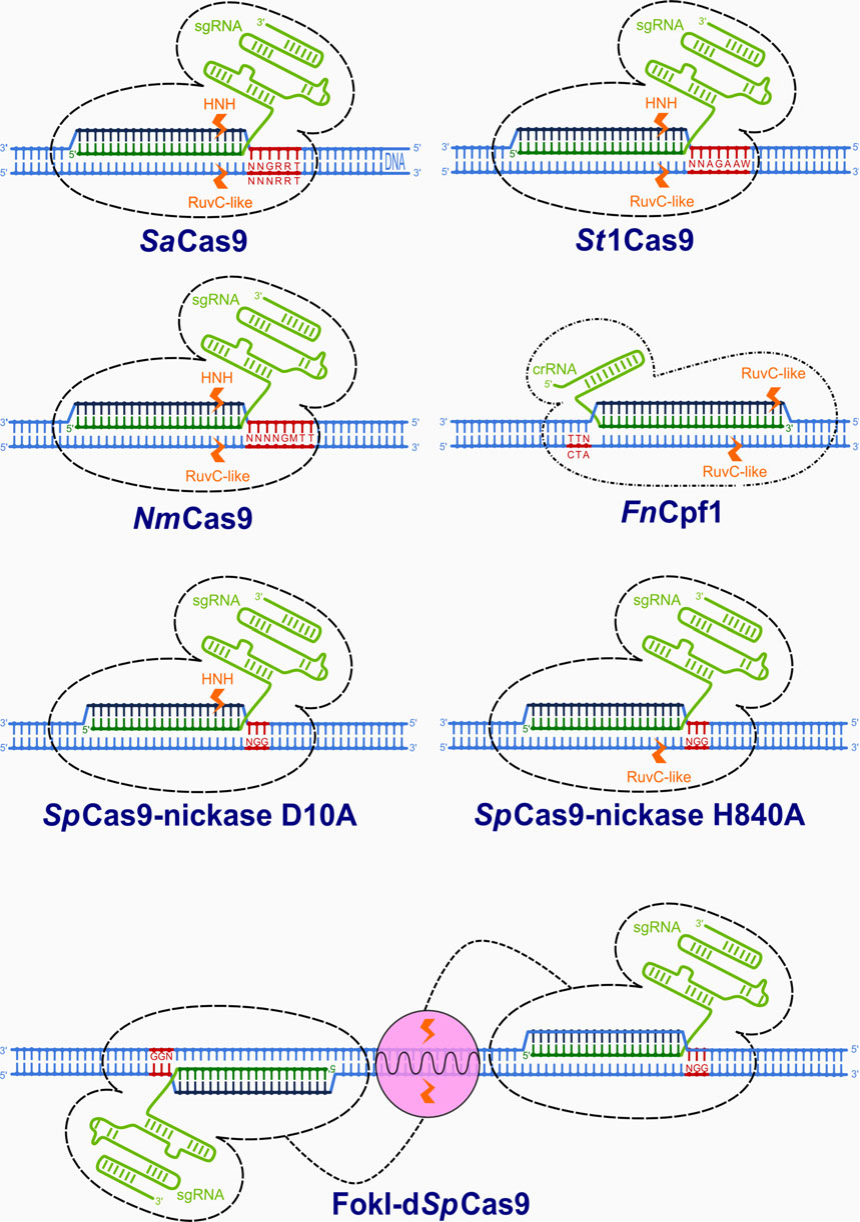
Different variants of DNA‐modifying enzymes derived from CRSIPR/Cas and CRISPR/Cpf1. Enzymes bind to DNA based on recognition of protospacer adjacent motifs (red) and Watson–Crick RNA–DNA pairing of complementary bases between DNA protospacers (dark blue) and sgRNA or crRNA guide sequences (dark green). Cleavage of single or both strands of DNA is performed by RuvC‐like, HNH or FokI nuclease domains (orange).

However, there are several other types of CRISPR RNA‐guided adaptive immune systems in microbial communities. They are divided into two major classes and further subdivided into five types and 16 subtypes (Makarova *et al*., 2015). Class 1 comprises three different types (Type I, Type III and Type IV) that rely on multi‐subunit protein complexes for crRNA binding, target binding and cleavage. Class 2 includes Type II, Type V and Type VI, all employing single effector proteins for binding crRNA and target, and for cleavage of target nucleic acids (Makarova *et al*., 2015). While the most widely known Class 2 Type II system is characterized by a single‐component effector protein, Cas9, with RuvC and HNH nuclease domains (Figure 1), the Class 2 Type V system utilizes a single RuvC domain containing effectors such as Cpf1 (Makarova *et al*., 2015; Zetsche *et al*., 2015; Figure 3), C2c1 or C2c3 (Shmakov *et al*., 2015); and Class 2 Type VI utilizes a single C2c2 effector with two higher eukaryotes and prokaryotes nucleotide‐binding (HEPN) RNase domains (Abudayyeh *et al*., 2016) (Figure 2). Some of these effectors have been successfully employed or have potential applications for gene and genome editing in eukaryotic cells, making them potentially useful in plant biotechnology. The aim of this review article is therefore to describe in detail these newly discovered CRISPR RNA‐guided effectors, their mode of action, some reported applications and to present their applicability as new tools for the plant science community.

## RNase activity—*Lsh*C2c2, *Fn*Cas9

With continuous fundamental research on basic mechanisms of bacterial immunity, several enzyme systems suitable for biotechnological applications have been discovered. An entirely new field of applications was opened up by the discovery of RNA dependent RNase enzyme systems from Class 2 Type II (*Fn*Cas9) and Class 2 Type VI (C2c2) immune systems.

Two recent publications characterize Class 2 type VI CRISPR effector C2c2 and describe its RNA‐guided RNase activity in bacteria (Abudayyeh *et al*., 2016; East‐Seletsky *et al*., 2016). Biochemical and *in vivo* analyses showed that C2c2 from the bacterium *Leptotrichia shahii* is guided by a single crRNA and can be programmed to cleave any ssRNA with complementary protospacers. C2c2 binding is determined by a crRNA secondary structure that must contain one stem–loop structure of at least 24‐nt and by a 22‐ to 28‐nt sequence complementary to the ssRNA protospacer (Figure 2). The latter must be flanked by a mononucleotide protospacer‐flanking site (PFS) at the 3′ end, preferentially composed of A, U or C. A mismatch‐sensitive ‘seed region’ exists in the centre of the crRNA–target duplex in which consecutive mismatches impede cleavage of target RNA despite C2c2 tolerance to single mismatches across the spacer. The C2c2 effector is composed of two HEPN domains with catalytic residues that cleave ssRNAs at varying distances outside the crRNA binding site (Figure 2), preferentially at uracil targets rather than to adenine targets. Unlike other CRISPR effector nucleases, C2c2, once primed by a cognate target, can also cleave other noncomplementary RNA molecules. Mutating the HEPN domains, by alanine substitution of any of the four predicted catalytic residues (R597A, H602A, R1278A, H1283A), produces catalytically inactive RNA‐binding proteins (d*Lsh*C2c2, analogous to d*Sp*Cas9), which can be used for RNA imaging in living cells (Abudayyeh *et al*., 2016; Rau and Rentmeister, 2016).

As of the time of writing, C2c2 has not yet been used in eukaryotic cells, but a similar RNase enzyme system, Cas9 from the Gram‐negative bacterium *Francisella novicida* (*Fn*Cas9), has already proved to be suitable for inhibition of human positive‐sense single‐stranded RNA (or (+)ssRNA) viruses in eukaryotic cells (Price *et al*., 2015). It was discovered in 2013 (Sampson *et al*., 2013) as an enzyme that targets bacterial mRNA, leading to alteration of gene expression. In 2015, it was engineered to target and destroy hepatitis C virus (HCV) in Huh‐7.5 cells, demonstrating that the method of RNA inhibition was very flexible in its targeting and was PAM‐independent. *Fn*Cas9 can target both positive‐sense and negative‐sense strands of RNA and inhibited RNA virus by blocking its translation and replication machinery. Interestingly, mutating the RuvC and HNH cleavage domains (D11A and H969A, respectively) of *Fn*Cas9 did not reduce inhibition of HCV, while mutation in the RNA‐binding arginine‐rich motif (ARM; R59A), which is necessary for the interaction of *Fn*Cas9 with nucleic acids, resulted in diminished HCV inhibition. Targeting of HCV showed that mismatches of up to six bases within the 3′ targeted region of the rgRNA (Figure 2) were tolerated without loss of HCV inhibition by *Fn*Cas9. Longer regions of mismatched bases at the 3′ or 5′ end, however, resulted in a loss of activity. Due to its cytosolic RNA targeting, the risk of an off‐target effect on host DNA seems to be limited, although *Fn*Cas9 is also capable of targeting DNA (Price *et al*., 2015).

These RNase enzyme systems hold great promise for engineering virus resistance and for regulation of gene expression in eukaryotic cells. As most plant viruses are composed of single‐stranded RNA genomes, we can shortly expect their application also for the development of plants tolerant of or resistant to viruses. The CRISPR/Cas9 platform from *Streptococcus pyogenes* has already been used for the development of plants resistant to several DNA viruses from the family Geminiviridae. Using transient assays and stable transformation of *Nicotiana benthamiana*, Baltes and colleagues (Baltes *et al*., 2015) demonstrated that *Sp*Cas9 can cleave the genome of a geminivirus as it spreads through the plant and therefore efficiently reduce virus load and symptoms by the formation of short (1–2 bp) indel mutations and by blocking Rep transcription, depending on the targeted region (Baltes *et al*., 2015). Simultaneously, CRISPR‐/Cas9‐induced geminivirus resistance was also reported in *Arabidopsis thaliana* and *N. benthamiana* (Ji *et al*., 2015). T1 plants with *Sp*Cas9 and sgRNAs elements integrated in their genomes showed an acquired CRISPR‐/Cas‐like immune system directed against geminoviruses, which was heritable by offspring. In a later study, Ali and colleagues (Ali *et al*., 2016) investigated the effect of targeting different monopartite (CLCuKoV, *Begomovirus*) and bipartite (MeMV, *Begomovirus*) geminiviruses, as well as different severe and mild strains of tomato yellow leaf curl virus (TYLCV, *Begomovirus*). The authors concluded that targeting intergenic and/or multiple regions of the virus genome simultaneously provides more effective strategies for virus management in plants (Ali *et al*., 2015a, 2016). Moreover, they demonstrated that it is possible to target more than one virus (TYLCV, BCTV and MeMV) with a single sgRNA (Ali *et al*., 2015a).

The knowledge obtained with *Sp*Cas9 against DNA viruses will help researchers in developing new biotechnological strategies for plant resistance against RNA viruses with the newly discovered RNase enzyme systems. Until now, genome editing in RNA virus control could only be used for targeting plant susceptibility genes. For example; to target ssRNA viruses in cucumber, *Sp*Cas9 was directed to eukaryotic translation initiator factor eIF4E and eIF(iso)4E, which is needed for translation of virus RNA. By mutating loci at N′ and C′ termini of eIF4E, researchers were able to obtain homozygous T3 cucumber lines immune to cucumber vein yellowing virus (CVYM, *Ipomovirus*,* Potyviridae*) and resistant to papaya ringspot virus‐W (PRSV‐W, *Potyvirus*,* Potyviridae*), as well as zucchini yellow mosaic virus (ZYMV, *Potyviridae*,* Potyvirus*) (Chandrasekaran *et al*., 2016). In *Arabidopsis*, complete resistance to turnip mosaic virus (TuMV, *Potyvirus*) was obtained with homozygous mutations in eIF(iso)4E (Pyott *et al*., 2016).

## Smaller size—*Sa*Cas9, *St1*Cas9, *Nm*Cas9, *Fn*Cpf1, *As*Cpf1, *Lb*Cpf1

As described above, the development of crop plants resistant to single or multiple viral infections, by targeting viral sequences, is feasible (Ali *et al*., 2015a) but it relies on the continuous presence and expression of Cas9 and sgRNAs (single or multiple) in plant genomes. In contrast, transgene elements needed for targeted mutagenesis of host susceptibility or other genes are needed only for a limited period of time and can be segregated out after mutagenesis. Heritable homozygous mutations can be therefore produced in the transgene‐free T2 generation in self‐compatible species, but the use of transgenesis during variety development can still trigger GMO regulation in countries that rely on process‐based regulatory approaches (Wolt *et al*., 2016).

To overcome this drawback, new, smaller variants of genome modifying enzymes are needed that can be used with viral vectors as the delivery method. Virus vectors enable high expression of heterologous genes without stable integration in host genomes and could therefore be very efficient for transient expression of genome editing systems. For example, tobacco rattle virus (TRV) vectors have already been used for targeted mutagenesis in *Nicotiana benthamiana* and *Petunia hybrida* genomes with zinc finger nucleases (Marton *et al*., 2010). As TRV systemically infects its host, it moved to and expressed ZFN also in the apical meristem and induced site‐specific mutations that were transmitted to the next generation (Marton *et al*., 2010). Such a nontransgenic approach for nuclease delivery and production of mutant plants does not require *in vitro* transformation and the regeneration step usually needed in genetic transformation. It could be therefore suitable for plant species recalcitrant to *in vitro* adventitious regeneration. However, so far, the technology cannot be used with *Sp*Cas9 due to its large size (4.2 kb), which exceeds TRV cargo capacities and has only been used for delivery and transcription of sgRNAs (Alagoz *et al*., 2016; Ali *et al*., 2015b) in plants that were overexpressing *Sp*Cas9 stably integrated into their genome (Ali *et al*., 2015a) or transiently expressed (Alagoz *et al*., 2016).

However, new possibilities could come with the recently described *Cas9* orthologous forms *Staphylococcus aureus* (*Sa*Cas9), *Streptococcus thermophilus* (*St*1Cas9) and *Neisseria meningitidis* (*Nm*Cas9), which are about 1 kb smaller (3.2, 3.4 and 3.2 kb, respectively) than *Sp*Cas9 (4.2 kb). They are all members of the Class 2 Type II immune system and act by double‐strand cleavage of invading DNA with the RuvC and HNH nuclease domains (Figure 3). *Sa*Cas9, *St*1Cas9 and *Nm*Cas9 induce DSB at specific customizable 21‐ to 24‐nt target sites near 5′‐NNGRRT‐3′ or 5′‐NNNRRT‐3′, 5′‐NNAGAAW‐3′ and 5′‐NNNNGMTT‐3′ PAM motifs, respectively (where N denotes any nucleotide, R purines A or G, M amino A or C and W weak interaction A or T; Table 1; Hou *et al*., 2013; Kleinstiver *et al*., 2015a,b; Ran *et al*., 2015).

**Table 1:** List of new variants of genome modifying enzyme systems with their main characteristics

Variant	First published	Novelty	Size (kb)	Addgene plasmid^a^	Published applications in plants
Class 2 type VI‐A CRISPR effector C2c2 (*Lsh*C2c2)	Abudayyeh *et al*. (2016) East‐Seletsky *et al*. (2016)	RNase function PFS ‐ C, A or U	~4.2	#79151	–
*Francisella novicida* Cas9 (*Fn*Cas9)	Sampson *et al*. (2013) Price *et al*. (2015)	RNase function PAM independent	~4.9	#68705	–
*Staphylococcus aureus* Cas9 (*Sa*Cas9)	Ran *et al*. (2015) Kleinstiver *et al*. (2015a,b)	Smaller size PAM ‐ NNGRRT, NNNRRT	~3.2	#61593	Kaya *et al*. (2016), Steinert *et al*. (2015)
*Streptococcus thermophilus* Cas9 (*St*1Cas9)	Kleinstiver *et al*. (2015b)	Smaller size PAM – NNAGAAW	~3.4	#65769	Steinert *et al*. (2015)
*Neisseria meningitidis* Cas9 (*Nm*Cas9)	Hou *et al*. (2013)	Smaller size PAM – NNNNGMTT	~3.2	#47867	‐
*Francisella novicida* Cpf1 (*Fn*Cpf1)	Zetsche *et al*. (2015)	Smaller size PAM ‐ TTN, CTA	~3.9	#69973	Endo *et al*. (2016)
*Acidaminococcus sp* Cpf1 (*As*Cpf1)	Zetsche *et al*. (2015)	Smaller size PAM – TTTN	~3.9	#69982	‐
*Lachnospiraceae bacterium* Cpf1 (*Lb*Cpf1)	Zetsche *et al*. (2015)	Smaller size PAM ‐ TTN	~3.7	#69988	Xu *et al*. (2016), Hu *et al*. (2016)
*Sp*Cas9‐nickase	Jinek *et al*. (2012) Ran *et al*. (2013)	Enhanced specificity	~4.1	#48873 D10A #79616 H840A	Fauser *et al*. (2014), Schiml *et al*. (2014), Mikami *et al*. (2016), Schiml *et al*. (2016)
e*Sp*Cas9	Slaymaker *et al*. (2016)	Enhanced specificity	~4.1	#71814	‐
Split‐*Sp*Cas9	Wright *et al*. (2015)	Smaller size Two‐component enzyme	~1.7 ~2.4	#62889	‐
d*Sp*Cas9‐FokI	Tsai *et al*. (2014) Guilinger *et al*. (2014)	Enhanced specificity	~4.8	#52970	‐
*Sp*Cas9‐cytidine deaminase	Komor *et al*. (2016) Nishida *et al*. (2016)	Enhanced specificity Gene editing without DSB	–	#73018 (BE1) #73019 (BE1) #73020 (BE2) #73021 (BE3)	Lu and Zhu (2016), Li *et al*. (2016)
d*Sp*Cas9‐gene expression functional domains	Qi *et al*. (2013)	Modulating gene expression	–	#44246	Piatek *et al*. (2015), Lowder *et al*. (2015)
d*Sp*Cas9‐Tet1 and ‐Dnmt3a	Liu *et al*. (2016), Vojta *et al*. (2016)	Editing CpG methylation	~6.4 ~6.9	#84475 (Tet1) #84476 (Dnmt3a)	‐

a Details about the plasmids can be found at : https://www.addgene.org/.

*Sa*Cas9 proved similar efficiencies in inducing mutagenesis in comparison with *Sp*Cas9 in human and mouse cells (Ran *et al*., 2015) and has already been used in tobacco (*Nicotiana tabacum*), rice (*Oryza sativa*) and *Arabidopsis thaliana* (Kaya *et al*., 2016; Steinert *et al*., 2015). In tobacco and rice, *Sa*Cas9 proved to have higher sequence recognition capacity than *Sp*Cas9, with multiple target sites and higher suitability for reducing off‐target mutations in crop species (Kaya *et al*., 2016). Using three of four possible *Sa*Cas9 PAM patterns at the 3′ end (5′‐NNGAGT‐3′, 5′‐NNGGGT‐3′, 5′‐NNGAAT‐3′), the researchers did not observe obvious differences in targeted mutagenesis efficiency and both G and A at the 4th or 5th positions of PAM were equally suitable for genome editing (Kaya *et al*., 2016). In *Arabidopsis thaliana*, Steinert and colleagues (Steinert *et al*., 2015) used codon‐optimized Cas9 from *Staphylococcus aureus* (*Sa*Cas9) and *Streptococcus thermophilus* (*St*1Cas9), both about 1 kb smaller (3.2 kb for *Sa*Cas9 and 3.4 kb for *St*1Cas9) than the commonly used *Sp*Cas9 (4.2 kb). They proved suitable for generation of heritable targeted mutagenesis events of the *ADH1* gene and stimulation of homologous recombination. For *St*1Cas9, both PAM motifs (5′‐NNAGAA‐3′ and 5′‐NNGGAA‐3′) were equally applicable and it cuts the DNA 2 or 3 nucleotides upstream of the PAM site (Steinert *et al*., 2015), similar to findings previously reported by Ran (Ran *et al*., 2015). *Sa*Cas9 cuts the DNA 4‐bases upstream of the PAM motif, and greater mutation yields were obtained with the 5′‐NNGGGT‐3′ PAM motif (up to 80%, most of them larger deletions) compared to the 5′‐NNGAAT‐3′ PAM motif (mostly single base pair insertions). The authors recommended 5′‐NNGGGT‐3′ PAM for gene editing with *Sa*Cas9 constructs, which additionally enabled induction of HR with an efficiency similar to that of *Sp*Cas9 in the reporter line used (Steinert *et al*., 2015).

Although the use of various CRISPR‐/Cas‐associated or fused enzyme activities in complex genetic approaches will be of special importance in the future (Steinert *et al*., 2015), the above‐described orthologs require longer PAMs than *Sp*Cas9, which decreases the number of potential target sites. Another group of recently discovered single crRNA‐guided DNase enzymes with shorter PAM motifs could be used instead. Class 2 Type V CRISPR effectors Cpf1, discovered in *Francisella novicida U112* (*Fn*Cpf1), *Acidaminococcus* sp. (*As*Cpf1) and *Lachnospiraceae bacterium* (*Lb*Cpf1), have already been successfully used in some eukaryotic cells (Zetsche *et al*., 2015), including rice and tobacco (Endo *et al*., 2016).

*Fn*Cpf1 utilizes a single short RNA guide molecule, 42‐ to 44‐nt crRNA, which begins with 19 nt of the direct repeat followed by 23–25 nt of the spacer sequence. *Fn*Cpf1 recognizes a short T‐rich (5′‐TTN‐3′) PAM upstream of the 5′ end and cleaves the DNA via a staggered DNA double‐stranded break after the 18th base on the nontargeted (+) strand and after the 23rd base on the targeted (−) strand. The induced indels are therefore located far from the seed region (the first five nucleotides on the 5′ end of the spacer sequence), which is thus preserved for subsequent cleavages. It generates 4‐ or 5‐nucleotide‐long 5′ overhangs. The generated ‘sticky ends’ at the cleavage sites may enhance the integration of DNA inserts (e.g. gene knockin) in the proper orientation also via a non‐HDR‐mediated repair mechanism. The Cpf1 protein contains a predicted RuvC‐like endonuclease domain, but it lacks the HNH endonuclease domain. Based on published results (Zetsche *et al*., 2015), the RuvC‐like domain of *Fn*Cpf1 cleaves both strands of the target DNA, both *in vitro* and *in vivo*. Further testing of 16 Cpf1‐family proteins from diverse bacteria, representing the entire Cpf1 diversity, revealed that only another two proteins, one from *Acidominococcus sp. BV3L6* (*As*Cpf1) and the other from *Lachnospiraceae bacterium ND2006* (*Lb*Cpf1), were efficient in genome editing of human embryonic kidney cells and showed comparable levels of indel formation to those of *Sp*Cas9. The remaining Cpf1 proteins showed only sporadic or no detectable activity at several tested loci, despite robust expression and comparable *in vitro* activities (Zetsche *et al*., 2015). The discrepancy between *in vitro* enzymatic activity and lack of *in vivo* editing ability might be attributable to the host chromatin context in the tested cell lines or their intrinsic property of those Cpf1 proteins. Nevertheless, it confirmed the previous results of Ran that only a small number of orthologs are successful for genome editing of human cells (Ran *et al*., 2015). Transgenic rice and tobacco plants constitutively expressing codon‐optimized *Fn*Cpf1 and crRNA showed targeted mutation in *N. benthamiana NtPDS* and *NtSTF1*, and rice *OsDL*,* OsALS*,* OsNCED1‐3*,* OsAO1‐5* loci. Mutations were observed at DNA (mostly deletions) and phenotype levels in transgenic tobacco and rice plants, as well as in some transgenic tobacco progenies, thus confirming that *Fn*Cpf1‐induced mutations are genetically transmitted to the next generation. Mutation efficiency was better in rice, with an average of 47.2% and in the creation of biallelic mutants in the T0 generation. In tobacco, the recorded average mutation frequency was 28.2% and no bialellic mutants were regenerated. Activity at off‐target sites was estimated in rice in *9‐cis‐epoxycarotenoid dioxygenase* (*NCED*) and *aldehyde oxidase* (*AO*) gene families. While the detected on‐target mutation frequency at *OsNCED1* was in the range 2.14%–23.3%, mutation frequencies at sites with one or two mismatched bases were 0%–6.25% and 0%, respectively. At the other gene loci, on‐target mutation frequencies at *OsAO1* and *OsAO2* were 38.8%–50% and 24.1%–36.6%, respectively, while this was 0%–5% at *OsAO4* with one mismatched base and (again) 0% at locus *OsAO5* with two mismatched bases (Endo *et al*., 2016).

## Enhanced specificity with *Sp*Cas9 variants—*Sp*Cas9‐nickase, split‐*Sp*Cas9, e*Sp*Cas9, *Sp*Cas9‐cytidine deaminase

Since the first biotechnological applications of *Sp*Cas9, many new variants of the enzyme have been developed. Most of the development has related to enhancing its specificity, as off‐target mutagenesis is still one of the major concerns for clinical applications and is of paramount importance in testing isogenic cell lines. *Sp*Cas9 specificity is also potentially important in crop plants with large genomes, often also polyploid, with many duplicated genes, which makes genome editing even more challenging.

Single‐stranded DNA cleavage *Sp*Cas9‐nickases were among the first *Sp*Cas9 variants to be obtained by mutations of one of two catalytic domains, HNH or RuvC (Gasiunas *et al*., 2012; Jinek *et al*., 2012). Single‐chain nicks are usually repaired by the high‐fidelity base excision repair pathway (BER) and are primarily used to facilitate homology‐directed repair (HDR) with minimal mutagenic activity. For obtaining DSB repaired by error‐prone nonhomologous end joining (NHEJ), *Sp*Cas9‐nickases are therefore used in pairs via appropriately offset (>100 bp long) guide RNAs (Ran *et al*., 2013). The strategy minimizes off‐target mutagenesis by extending the length of the recognized DNA target region from 23 bp to 2 × 23 bp (Figure 3), while maintaining on‐target cleavage rates similar to those of wild‐type *Sp*Cas9. It was demonstrated that using paired nickases can reduce off‐target activity by 50‐ to 1500‐fold in human cell lines without diminishing on‐target cleavage efficiency (Ran *et al*., 2013). In *Arabidopsis*, single *Sp*Cas9 D10A nickase did not produce detectable error‐prone NHEJ events but was as efficient as nuclease or homing endonuclease I, SceI, in stimulating homologous recombination (Fauser *et al*., 2014). In contrast, paired *Sp*Cas9 nickases had mutagenic efficiency comparable to that of the *Sp*Cas9 nuclease and the obtained mutations were mostly deletions, while insertions were detected at lower frequencies. While *Sp*Cas9 nuclease mainly caused 1‐bp insertions, the insertions produced by paired nickases were considerably longer and their sequences originated mostly from the vicinity of the insertion site (Schiml *et al*., 2014). The authors assumed that the obtained double‐strand breaks in the *ADH1* gene, with 52‐nucleotide‐long DNA single‐stranded overhangs, were processed differently than DSBs with short or without overhangs (Schiml *et al*., 2014).

Similar results were obtained in rice plants regenerated from treated calli, where paired *Sp*Cas9 nickases did not produce detectable off‐target mutations at paralogue genes *s*,* OsCDKA2*,* OsCDKB1* and *OsCDKA1*. Moreover, combinations of sgRNAs containing only a single nucleotide mismatch to the DNA target sequence could not induce mutations (Mikami *et al*., 2016). Unfortunately, in rice, the on‐target mutation (at *OsDMC1A* and *OsCDKB2* genes) frequency was also lower to different extents in comparison with *Sp*Cas9 nuclease, depending on the sgRNA used (Mikami *et al*., 2016). These results suggested that 3′ overhang structures obtained with paired nickases induce fewer mutations compared to 5′ overhang structures, probably by favouring HDR (Mikami *et al*., 2016), which was in accordance with the results obtained at three genomic loci in human cells (Ran *et al*., 2013).

With structure‐guided protein engineering, Slaymaker and colleagues (Slaymaker *et al*., 2016) improved the specificity of *Sp*Cas9 by attenuating its helicase activity, and developed an enhanced version of *Sp*Cas9 (e*Sp*Cas9). They neutralized positively charged residues within the nontarget strand groove, which is positioned between the HNH, RuvC and PAM‐interacting domains and is involved in stabilizing the nontarget strand of the target DNA. They hypothesized that in such mutated *Sp*Cas9, mismatches between sgRNA and target DNA would be less energetically favourable, which would lead to reduced cleavage activity at off‐target sites. The results confirmed their hypothesis and showed reduced off‐target effects while maintaining robust on‐target cleavage at several target sites in HEK cells when using single amino acid mutants of *Sp*Cas9 or combination mutants. Off‐target indels for a wide array of different sgRNAs could not be detected, even using a highly sensitive genomewide sequencing method (Slaymaker *et al*., 2016).

Another approach for enhancement of *Sp*Cas9 specificity was created by Wright and colleagues (Wright *et al*., 2015), who developed a binary *Sp*Cas9 system (split‐*Sp*Cas9) by overexpressing the nuclease lobe and α‐helical lobe as separate polypeptides in *Escherichia coli*. The lobes were not able to interact on their own, but their activity was restored by binding sgRNA, which acted as a molecular scaffold for dimerization of the lobes. Dimerization was disabled by the removal of one or more hairpins at the 3′ end of sgRNA. The split‐*Sp*Cas9 complex was able to catalyse site‐specific cleavage of DNA *in vitro* at efficiencies comparable to those of wild‐type *Sp*Cas9, with the advantage of being regulatable. The split‐*Sp*Cas9 polypeptides were nucleofected in HEK293T cells with sgRNA targeting the *EMX1* locus. Compared to wild type, reduced levels of indels were presumably attributable to ternary complex disruption during dilution and nucleofection, and slower kinetics of DNA cleavage in cells by the split‐*Sp*Cas9. The authors postulated a potential application of split‐*Sp*Cas9 with viral‐mediated delivery with limited packaging capacity (Wright *et al*., 2015). A method with adeno‐associated viral delivery was later successfully tested in mice (Chew *et al*., 2016).

A different approach to specificity enhancement was used by Komor *et al*. (2016), who fused *Sp*Cas9‐nickase with cytidine deaminase and created *Sp*Cas9‐CD. While all other *Sp*Cas9 variants rely on inducing DBS to add, remove or change the DNA sequence, this variant mediates the direct conversion of cytidine to uridine, which has the base pairing properties of thymine (T). Upon DNA replication, adenine (A) binds to U, and T to A, resulting in a C→T (or G→A) substitution. The enzyme itself is made of Uracil DNA glycosylase inhibitor (UGI) and APOBEC rat deaminase, fused to N‐terminus of *Sp*Cas9‐nickase with a 3‐ to 21‐amino acid‐long XTEN linker. This results in ‘base editing’ machinery that can efficiently convert cytidines within a five‐nucleotide window between position 4 and 8 at the 3′ end of PAM. It works by transforming cytidine to uracil and nicking the opposite strand of the targeted sequence causing eukaryotic mismatch repair to help resolve the U:G mismatch into the desired U:A or T:A product. Three variants of the protein have been created and tested (BE1, BE2, BE3), but BE3 has proved itself most efficient compared to the others. The results revealed that base editing can be much more efficient in DNA editing of single bases in human cells than *Sp*Cas9‐mediated HDR and with substantially less or no indel formation (Komor *et al*., 2016).

## Dead *Sp*Cas9 fused with different functional domains ‐ FokI‐d*Sp*Cas9, d*Sp*Cas9‐EDLL, d*Sp*Cas9‐TAD, d*Sp*Cas9‐SRDX, d*Sp*Cas9‐Tet1 and d*Sp*Cas9‐Dnmt3a

Mutations of both cleavage domains of *Sp*Cas9 (D10A for RuvC and H840A for HNH) result in so‐called dead Cas9 (d*Sp*Cas9), an RNA‐guided DNA binding protein without cleavage activity (Jinek *et al*., 2012). Fused with fluorescent or other types of marker, it can be used in basic research for live DNA imaging or in biotechnological applications when fused with other functional domains.

One such is the fusion of catalytically inactive, ‘dead’ *Sp*Cas9 with the FokI nuclease domain at the N‐terminus (FokI‐dCas9; Tsai *et al*., 2014). The nonspecific DNA cleavage domain of FokI has been extensively used for genome editing with zinc finger nucleases (ZFN) and transcription activator‐like effector nucleases (TALEN) in the past. FokI can cleave any DNA sequence, the site of cleavage being determined by DNA binding domains (proteins in ZFN and TALEN, RNA in Cas9; Weeks *et al*., 2016). As the FokI domain is active only as a homodimer and is therefore more precise than monomeric *Sp*Cas9, fewer off‐target mutations can be expected. Indeed, much of the greater specificity gained from using a pair of d*Sp*Cas9‐FokI molecules is that 46 nucleotides (2 × 23 bp) are involved in target site recognition. As revealed by EGFP disruption assay in human cell lines, to induce DSB, two FokI monomers must bind to DNA and dimerize with an optimal spacing of 14–17 nucleotides distal to PAM (Figure 3) in order to enable efficient cleavage (Tsai *et al*., 2014). The requirement for simultaneous co‐localization of two FokI monomers at a defined distance reduces the chances of finding more than one suitable target site in the genome and thus enhances specificity even more than paired *Sp*Cas9‐nickases. Experiments on two human cell lines demonstrated that FokI‐d*Sp*Cas9 is efficient in modifying several endogenous human genes and that it can eliminate *Sp*Cas9‐induced off‐target effects of single sgRNAs (Tsai *et al*., 2014). The authors also observed that *Sp*Cas9‐nickase induces a higher rate of off‐target indel mutations than FokI‐d*Sp*Cas9 when both are directed by the same single sgRNA and that monomeric *Sp*Cas9‐nickase can in some cases induce unwanted base pair substitutions into their target sites, which was previously unknown (Tsai *et al*., 2014).

Nowadays, CRISPR/Cas9 technology can also enable spatiotemporal modulation of gene transcript levels using d*Sp*Cas9 alone (so‐called CRISPR interference or CRISPRi technology; Qi *et al*., 2013) or fused with appropriate gene transcription activation or repression domains (Perez‐Pinera *et al*., 2013). In 2015, there was the first publication of d*Sp*Cas9 fused with transcription regulators (Piatek *et al*., 2015), in which the authors generated synthetic transcriptional activators by fusing the d*Sp*Cas9 C‐terminus to the EDLL domain or the TAL activation domain (TAD) and created d*Sp*Cas9:EDLL and d*Sp*Cas9:TAD synthetic transcriptional activators. The experiment was performed as a transient expression system by Agro‐infiltration of *Nicotiana benthamiana* leaves with effector, guide RNA and target molecules. The hybrid proteins were directed towards the promoter regions of Bs3::uidA and the promoter or first exon of the *phytoene desaturase* gene (*PDS*). No stably transformed lines were regenerated but the results confirmed EDLL and TAD as strong transcriptional activators in plants, even with human codon‐optimized d*Sp*Cas9. A 14‐fold increase in transcriptional activation was detected by RT‐qPCR and a five‐ to sixfold by quantitative GUS assay.

Synthetic repressors have also been developed, by binding a SRDX repression domain from the ERF transcription factor to d*Sp*Cas9. When trying to block gene expression using only d*Sp*Cas9 with sgRNAs targeting sense and antisense strands in the promoter region of the *PDS* gene or the sense strand of the first exon, markedly reduced *PDS* transcript levels were obtained compared to control conditions. Additionally, an additive effect on the reduction in transcription was observed when all three sgRNAs were used simultaneously, and similar results were obtained with d*Sp*Cas9:SRDX. By co‐delivery of synthetic activator and synthetic repressor constructs, the authors observed that d*Sp*Cas9:SRDX interferes with transcriptional activation by both synthetic activators (d*Sp*Cas9:EDLL and d*Sp*Cas9:TAD). They demonstrated the versatility and reproducibility of the modified d*Sp*Cas9 system for targeted gene activation and repression in plants, for both heterologous reporter genes and endogenous genes (Piatek *et al*., 2015). Expression plasmids for regulation of gene expression, with VP64 transcriptional activator or SRDX transcriptional repressor, can easily be cloned with the use of Gateway vectors developed by Lowder and colleagues, available at Addgene, Kerafast and Arabidopsis Biological Resource Center (ABRC) (Lowder *et al*., 2015).

Modulation of gene expression can also be obtained by altering the methylation patterns of promoters with catalytically inactive *Sp*Cas9 fused with methylases and/or demethylases, as demonstrated in mammalian cells with d*Sp*Cas9‐Tet1 and d*Sp*Cas9‐Dnmt3a (Liu *et al*., 2016). Targeting of such fusion proteins to a methylated or unmethylated promoter of an endogenous gene sequence caused activation or silencing, respectively. This system was successfully used in *in vivo* experiments on mice, which confirmed the feasibility of the technology for functional studies of epigenetic regulation (Liu *et al*., 2016). In plants, epigenetic regulation involving DNA methylation and demethylation plays important roles in manifestation of gene function in plant immunity against pathogens, abiotic stress responses, heterosis, environmental memory and others (Law and Jacobsen, 2010). Programmed removal or addition of methyl groups from regulatory regions using engineered CRISPR/Cas systems enables alteration in gene expression (induction or suppression) of genes involved in these biological processes. The technology would benefit basic understanding of epigenetics in complex traits and also provide new strategies for enhancing the performance of crop plants.

## Conclusions

The new enzyme systems presented in this review article widen the toolbox for plant genome engineering and could accelerate research in the fields of plant biotechnology and breeding, as well as in basic plant science. In the future, these systems could enable many powerful applications, including targeted mutagenesis in plant cells without the need of transgenesis (e.g. using pre‐assembled ribonucleoproteins (purified *Sp*Cas9 protein + *in vitro* transcribed or synthetic sgRNA), as has been demonstrated in *Arabidopsis*, tobacco, lettuce, rice (Woo *et al*., 2015), petunia (Subburaj *et al*., 2016), maize (Svitashev *et al*., 2016), apple and grape (Malnoy *et al*., 2016), rice and soya bean (Kim *et al*., 2017), and wheat (Liang *et al*., 2017); stimulation of several different catalytic functions simultaneously in a single cell by combining different enzyme systems; reprogramming complex transcriptome patterns of plant cells; multicolour imaging of plant chromosomes by the usage of several fluorescently labelled catalytically inactive orthologs and many others. Moreover, some of these new systems could be used for conventional mutations or HDRs of targets that lack *Sp*Cas9 PAM sequence 5′‐NGG‐3′. Based on positive results obtained in a number of eukaryotic cells, we believe that these new variants also have great potential in plant sciences. Their implementation depends on their future performance and on new variants that will probably still emerge. Indeed, as recently reported, genome‐resolved metagenomics analysis has revealed that a plethora of other genome modifying enzyme systems exist in uncultivated bacteria and archaea (Burstein *et al*., 2017).

## Conflict of interest

The authors have no conflict of interest to declare.
